# Grammatical-Restrained Hidden Conditional Random Fields for Bioinformatics applications

**DOI:** 10.1186/1748-7188-4-13

**Published:** 2009-10-22

**Authors:** Piero Fariselli, Castrense Savojardo, Pier Luigi Martelli, Rita Casadio

**Affiliations:** 1 Biocomputing Group, University of Bologna, via Irnerio 42, 40126 Bologna, Italy

## Abstract

**Background:**

Discriminative models are designed to naturally address classification tasks. However, some applications require the inclusion of grammar rules, and in these cases generative models, such as Hidden Markov Models (HMMs) and Stochastic Grammars, are routinely applied.

**Results:**

We introduce Grammatical-Restrained Hidden Conditional Random Fields (GRHCRFs) as an extension of Hidden Conditional Random Fields (HCRFs). GRHCRFs while preserving the discriminative character of HCRFs, can assign labels in agreement with the production rules of a defined grammar. The main GRHCRF novelty is the possibility of including in HCRFs prior knowledge of the problem by means of a defined grammar. Our current implementation allows *regular grammar *rules. We test our GRHCRF on a typical biosequence labeling problem: the prediction of the topology of Prokaryotic outer-membrane proteins.

**Conclusion:**

We show that in a typical biosequence labeling problem the GRHCRF performs better than CRF models of the same complexity, indicating that GRHCRFs can be useful tools for biosequence analysis applications.

**Availability:**

GRHCRF software is available under GPLv3 licence at the website

## Background

Sequence labeling is a general task addressed in many different scientific fields, including Bioinformatics and Computational Linguistics [1-3]. Recently Conditional Random Fields (CRFs) have been introduced as a new promising framework to solve sequence labeling problems [4]. CRFs offer several advantages over Hidden Markov Models (HMMs), including the ability of relaxing strong independence assumptions made in HMMs [4]. CRFs have been successfully applied in biosequence analysis and structural predictions [5-11]. However, several problems of sequence analysis can be successfully addressed only by designing a grammar in order to provide meaningful results. For instance in gene prediction tasks exons must be linked in such a way that the donor and acceptor junctions define regions whose length is multiple of three (according to the genetic code), and in protein structure prediction, helical segments shorter than 4 residues should be consider meaningless, being this the shortest allowed length for a protein helical motif [1,2]. In this kind of problems, the training sets generally consist of pairs of observed and label sequences and very often the number of the different labels representing the experimental evidence is small compared to the grammar requirements and the length distribution of the segments for the different labels. Then a direct mapping of one-label to one state results in poor predictive performances and HMMs trained for these applications routinely separate labels from state names. The separation of state names and labels allows to model a huge number of concurring paths compatible with the grammar and with the experimental labels without increasing the time and space computational complexity [1].

In analogy with the HMM approach, in this paper we develop a discriminative model that incorporates regular-grammar production rules with the aim of integrating the different capabilities of generative and discriminative models. In order to model labels and states disjointly, the regular grammar has to be included in the structure of a Hidden Conditional Random Field (HCRF) [12-14]. Previously, McCallum et al. [13] introduced a special HCRF that exploits a specific automaton to align sequences.

The model here introduced as Grammatical-Restrained Hidden Conditional Random Field (GRHCRF), separates the states from the labels and restricts the accepted predictions only to those allowed by a predefined grammar. By this, it is possible to cast into the model prior knowledge of the problem at hand, that may not be captured directly from the learning associations and ensures that only meaningful solutions are provided.

In principle CRFs can directly model the same GRHCRF grammar. However, given the fully-observable nature of the CRFs [12], the observed sequences must be re-labelled to obtain a bijection between states and labels. This implies that only one specific and *unique *state path for each observed sequence must be selected. On the contrary with GRHCRFs that allow the separation between labels and states, an arbitrary large number of different state paths, corresponding to the same experimentally observed labels, can be counted at the same time. In order to fully exploit this path degeneration in the prediction phase, the decoding algorithm must take into account all possible paths, and the posterior-Viterbi (instead of the Viterbi) should be adopted [15].

In this paper we define the model as an extension of a HCRF, we provide the basic inference equations and we introduce a new decoding algorithm for CRF models. We then compare the new GRHCRF with CRFs of the same complexity on a Bioinformatics task whose solution must comply with a given grammar: the prediction of the topological models of Prokaryotic outer membrane proteins. We show that in this task the GRHCRF performance is higher than to those achieved by CRF and HMM models of the same complexity.

## Methods

**Figure 1:**
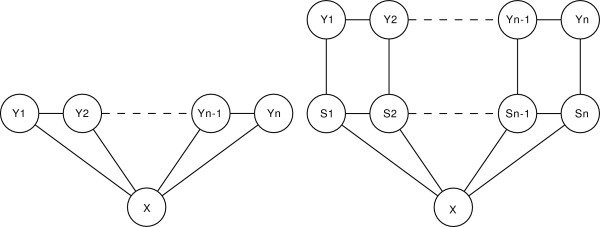
**Graphical structure of a linear-CRF (left) and a linear GRHCRF/HCRF (right)**.

In what follows **x **is the random variable over the data sequences to be labeled, **y **is the random variable over the corresponding label sequences and **s** is the random variable over the hidden states. We use an upper-script index when we deal with multiple sequences. The problem that we want to model is then described by the observed sequences **x**^(*i*)^, by the labels **y**^(*i*) ^and by the underlying grammar *G *that is specified by its production rules with respect to the set of the hidden states. Although it is possible to imagine more complex models, in what follows we restrict each state to have only one possible associated label. Thus we define a function that maps each hidden state to a given label as:



The difference between the CRF and GRHCRF (or HCRF) models can be seen in Figure 1, where their graphical structure is presented. GRHCRF and HCRF are indistinguishable from their graphical structure representation since it depicts only the conditional dependence among the random variables. Since the number of the states |{**s**}| is always greater than the number of possible labels |{**y**}| the GRHCRFs (HCRFs) have more expressive power than the corresponding CRFs.

We further restrict our model to linear HCRF, so that the computational complexity of the inference algorithms remains linear with respect to the sequence length. This choice implies that the embedded grammar will be *regular*. Our implementation and tests are based on first order HCRFs with explicit transition functions (*t*_*k*_(*s*_*j*-1_, *s*_*j*_, **x**)) and state functions (*g*_*k*_(*s*_*j*_, **x**)) unrolled over each sequence position *j*.

However, for sake of clarity in the following we use the compact notation:



where *f*_*k*_(*s*_*j*-1_, *s*_*j*_, **x**) can be either a transition feature function *t*_*l*_(*s*_*j*-1_, *s*_*j*_, **x**) or a state feature function *gn*(*s*_*j*_, **x**). Following the usual notation [16] we extend the local functions to include the hidden states as

(1)

and we set the two constraints as:



With this choice, the local function ψ_*j*_(**s**, **y**, **x**) becomes zero when the labeling (Ω(*s*_*j*_, *y*_*j*_)) or the grammar production rules (Γ(*s*, *s*')) are not allowed. In turn this sets to zero the corresponding probabilities. As in the case of the HCRF, for the whole sequence we define Ψ(**s**, **y**, **x**) = Π_*j*_ψ_*j*_(**s**, **y**, **x**) and the normalization factors (or partition functions) can be obtained summing over all possible sequences of hidden states (or latent variables):



or summing over all possible sequences of labels and hidden states:



Using the normalization factors the joint probability of a label sequence **y **and an hidden state sequence **s **given an observation sequence **x **is:



The probability of an hidden state sequence given a label sequence and an observation sequence is:



Finally, the probability of a label sequence given an observation sequence can be computed as follows:



### Parameter estimation

The model parameters (θ) can be obtained by maximizing the log-likelihood of the data:



where the different sequences are supposed to be independent and identically distributed random variables.

Taking the first derivative with respect to parameter λ_*k *_of the objective function we obtain:



where, in analogy with the Boltzmann machines and HMMs for labelled sequences [17],  and ℱ an be seen as *clamped *and *free *phases. After simple computations we can rewrite the derivative as:



where the *E*_*p*(**s**|**y**, **x**) _[*f*_*k*_] and *E*_*p*(**s**, **y**|**x**) _[*f*_*k*_] are the expected values of the feature function *f*_*k *_computed in the clamped and free phases, respectively. Differently from the standard CRF, both expectations have to be computed using the Forward and Backward algorithms. These algorithms must take into consideration the grammar restraints.

To avoid overfitting, we regularize the objective function using a Gaussian prior, so that the function to maximize has the form of:



and the corresponding gradient is:



Alternatively, the Expectation Maximization procedure can be adopted [16].

### Computing the expectations

The partition functions and the expectations can be computed using the dynamic programming by defining the so called forward and backward algorithms [1,2,4]. For the clamped phase the forward algorithm is:



where the clamped phase matrix *M*_*C *_takes into account both the grammar constraint (Γ(*s*', *s*)) and the current given labeling **y **.



The forward algorithm for the free phase is computed as:



where the free phase matrix *M*_*F *_is defined as:



It should be noted that also in the free phase the algorithm has to take into account the grammar production rules Γ(*s*', *s*) and only the paths that are in agreement with the grammar are counted. Analogously, the backward algorithms can be computed for the clamped phase as:



where *L*^(*i*) ^is the length of the *i*^*th *^protein. For the free phase we have:



The expectations of the feature functions (*E*_*p*(**s**|**y**, **x**) _[*f*_*k*_], *E*_*p*(**s**, **y**|**x**) _[*f*_*k*_]) are computed as:



The partition functions can be computed using both forward or backward algorithms as:



where for simplicity we dropped out the sequence upper-script ((*i*)).

### Decoding

Decoding is the task of assigning labels (**y**) to an unknown observation sequence **x**. Viterbi algorithm is routinely applied as decoding for the CRFs, since it finds the most probable path of an observation sequence given a CRF model [4]. Viterbi algorithm is particular effective when there is a single strong highly probable path, while when several paths compete (have similar probabilities), *posterior decoding *may perform significantly better. However, the selected state path of the posterior decoding may not be allowed by the grammar. A simple solution of this problem is provided by the posterior-Viterbi decoding, that was previously introduced for HMMs [15]. Posterior-Viterbi, exploits the posterior probabilities and at the same time preserves the grammatical constraint. This algorithm consists of three steps:

• for each position *j *and state *s *∈ , compute posterior probability *p*(*s*_*j *_= *s*|**x**)

• find the allowed state path

S* = argmax_**s **_Π_*j *_*p*(*s*_*j *_= **s**|**x**)

• assig to **x **a label sequence **y **so that *y*_*j *_= Λ(*s*_*j*_) for each position *j*

The first step can be accomplished using the Forward-Backward algorithm as described for the free phase of parameter estimation. In order to find the best allowed state path, a Viterbi search is performed over posterior probabilities. In what follows *ρ*_*j*_(*s*|**x**) is the most probable allowed path of length *j *ending in state *s *and *π*_*j*_(*s*) is a traceback pointer. The algorithm can be described as follows:

1. Initialization:



2. Recursion



3. Termination and Traceback



The labels are assigned to the observed sequence according to the state path s*. It is also possible to consider a slightly modified version of the algorithm where, for each position, the posterior probability of the *labels *is considered, and the states with the same label have associated the same posterior probability. The rationale behind this is to consider the aggregate probability of all state paths corresponding to the same sequence of labels to improve the overall per label accuracy. In many applications this variant of the algorithm might perform better.

### Implementation

We implemented the GRHCRF as linear HCRF in C++ language. Our GRHCRF can deal with sequences of symbols as well as sequence profiles. A *sequence profile *of a protein *p *is a matrix *X *whose rows represent the sequence positions and whose columns are the 20 possible amino acids. Each element *X *[*i*] [*a*] of the sequence profile represents the frequency of the residue type *a *in the aligned position *i*. The profile positions are normalized such as Σ_*a*_*X*[*i*][*a*] = 1 (for each *i*).

In order to take into account the information of the neighboring residues we define a symmetric sliding window of length *w *centered into the *i*-th residue. With this choice the state feature functions are defined as:



where *s *runs over all possible states, *a *runs over the different observed symbols *A *(in our case the 20 residues) and *k *runs over the neighbor residues (from -  to ). When dealing with single sequences, the state functions are simply products of Kronecker's deltas:



while in the case of sequence profiles, the state features are real-valued and assume the profile scores:



## Measures of performance

To evaluate the accuracy we define the classical label-based indices, such as:



where *p *and *N *are the total number of correct predictions and total number of examples, respectively. The Matthews correlation coefficient (C) for a given class s is defined as:



*p*(*s*) and n(*s*) are respectively the true positive and true negative predictions for class *s*, while *o*(*s*) and *u*(*s*) are the numbers of false positives and false negatives with respect to that class. The sensitivity (coverage, *Sn*) for each class *s *is defined as



The specificity (accuracy, *Sp*) is the probability of correct predictions and it is defined as follows:



However, these measures cannot discriminate between similar and dissimilar segment distributions and do not provide any clues about the number of proteins that are correctly predicted. For this reason we introduce a *protein-based *index, the Protein OVerlap (POV) measure. We consider a protein prediction to be correct only if the number of predicted and observed transmembrane segments (in the structurally resolved proteins, see Outer-membrane protein data set section) is the same and if all corresponding pairs have a minimum segment overlap. POV is a binary measure (0 or 1) and for a given protein sequence *s *is defined as:



Where  and  are the numbers of predicted and observed segments, while *p*_*i *_and *o*_*i *_are the *i*^*th *^predicted and observed segments, respectively. The threshold θ is defined as the mean of the half lengths of the segments:



where *L*_*p*_(= |*p*_*i*_|) and *L*_*o*_(= |*o*_*i*_|) are the lengths of the predicted and observed segments, respectively. For a set of proteins the average of all POVs over the total number of proteins *N *is:



To evaluate the average standard deviation of our predictions, we performed a bootstrapping procedure with 100 runs over 60% of the predicted data sets.

## Results and Discussion

### Problem definition

The prediction of the topology of the outer membrane proteins in Prokaryote organisms is a challenging task that was addressed several times given its biological relevance [18-20]. The problem can be defined as: given a protein sequence that is known to be inserted in the outer membrane of a Prokaryotic cell, we want to predict the number and the location with respect to the membrane plane of the membrane-spanning segments. From experimental results, we know that the outer membrane of Prokaryotes imposes some constraints to the topological models such as:

• both C and N termini of the protein chain lie in the periplasmic side of the cell (inside) and this implies that the number of the spanning segments is even;

• membrane spanning segments have a minimal segment length (≥ 3 residues);

• the transmembrane-segment lengths are distributed accordingly to a probability density distribution that can be experimentally determined and must be taken into account.

For the reasons listed above the best performing predictors described in literature are based on HMMs and among them the best performing single-method in the task of the topology prediction is HMM-B2TMR [18] (see Table 1 in [20]).

**Table 1:** Prediction of the topology of the Prokaryotic outer membrane proteins.

**Method**	**POV**	**Q2**	**C(t)**	**Sn(t)**	**Sp(t)**
CRF-1 (Vit)	0.26 ± 0.05	0.72 ± 0.01	0.47 ± 0.02	0.59 ± 0.01	0.80 ± 0.01
CRF-1 (Pvit)	0.39 ± 0.05	0.77 ± 0.01	0.54 ± 0.02	0.71 ± 0.01	0.80 ± 0.01
					
CRF-2 (Vit)	0.34 ± 0.05	0.76 ± 0.01	0.52 ± 0.03	0.63 ± 0.02	0.82 ± 0.02
CRF-2 (Pvit)	0.47 ± 0.05	0.80 ± 0.01	0.60 ± 0.03	0.74 ± 0.02	0.82 ± 0.02
					
CRF-3 (Vit)	0.29 ± 0.04	0.72 ± 0.01	0.45 ± 0.02	0.60 ± 0.02	0.79 ± 0.01
CRF-3 (Pvit)	0.45 ± 0.04	0.76 ± 0.01	0.52 ± 0.02	0.70 ± 0.02	0.79 ± 0.01
					
GRHCRF	0.66 ± 0.04	0.85 ± 0.01	0.70 ± 0.03	0.83 ± 0.01	0.84 ± 0.01
					
HMM-B2TMR	0.58 ± 0.04	0.80 ± 0.01	0.62 ± 0.02	0.82 ± 0.02	0.83 ± 0.01

C(t), Sn(t) and Sp(t) are reported for the transmembrane segments (t).

Vit = Viterbi decoding, Pvit = posterior-Viterbi decoding.

For GRHCRF and HMM-B2TMR we used the posterior-Viterbi decoding.

Models are detailed in the text. Scoring indices are described in Measure of Accuracy section.

### Outer-membrane protein data set

The training set consists of 38 high-resolution experimentally determined outer-membrane proteins of Prokaryotes, whose sequence identity between each pair is less than 40%. We then generated 19 subsets for the cross-validation experiments, such as there is no sequence identity greater than 25% and no functional similarity between two elements belonging to disjoint sets. The annotation consists of three different labelings that correspond to: *inner loop *(i), *outer loop *(o) and *transmembrane *(t). This assignment was obtained using the DSSP program [21] by selecting the *β*-strands that span the outer membrane. The dataset with the annotations and the cross-validation sets are available with the program at .

For each protein in the dataset, a profile based on a multiple sequence alignment was created using the PSI-BLAST program on the non-redundant dataset of sequences (uniref90 as described in ). PSI-BLAST runs were performed using a fixed number of cycles set to 3 and an e-value of 0.001.

### Prediction of the topology of Prokaryotic outer membrane proteins

**Figure 2:**
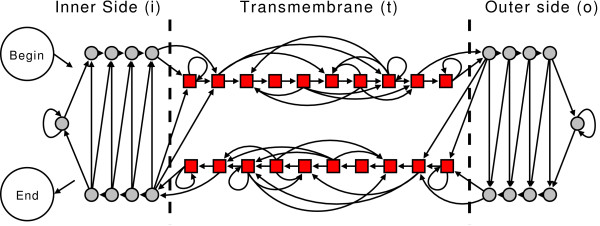
**Automaton structure designed for the prediction of the topology of the outer-membrane proteins in Prokaryotes with GRHCRFs and HMMs**.

The topology of outer-membrane proteins in Prokaryotes can be described assigning each residue to one of three types: inner loop (*i*), transmembrane *β*-strand (*t*), outer loop (*o*). These three types are defined according to the experimental evidence and are the *terminal *symbols of the grammar. The chemico-physical and geometrical characteristics of the three types of segments as deduced by the available structures in the PDB suggest how to build a grammar (or the corresponding automaton) for the prediction of the topology. We performed our experiments using the automaton depicted in Figure 2, which was previously introduced to model our HMM-B2TMR [18] (this automaton is substantially similar to all other HMMs used for this task [19,20]). It is essentially based on three different types of states. The states of the automaton are the *non-terminal *symbols of the regular grammar and the arrows represent the allowed transitions (or production rules). The states represented with squares describe the transmembrane strands while the states shown with circles represent the loops (Figure 2). A statistics on the non-redundant database of outer membrane proteins presently available, indicates that the length of the strands of the training set ranges from 3 to 22 residues (with an average length of 12 residues). In Prokaryotic outer membrane proteins the inner loops are generally shorter than outer loops. Furthermore, both the N-terminus and C-terminus of all the proteins lie in the inner side of the membrane [18]. These constraints are modelled by means of the allowed transitions between the states.

The automaton described in Figure 2 assigns labels to observed sequences that can be obtained using different state paths. This ambiguity leads to an ensemble of paths that must be taken into account during the likelihood maximization by summing up all possible trajectories compliant with the experimentally assigned labels (see Method section).

However, this ambiguity does not permit the adoption of the automaton of Figure 2 for CRF learning, since to train CRFs a bijective mapping between states and labels is required. On the contrary, with the automaton of Figure 2, several different state paths can be obtained (in theory a factorial number) that are in agreement with the automaton and with the experimental labels.

**Figure 3:**
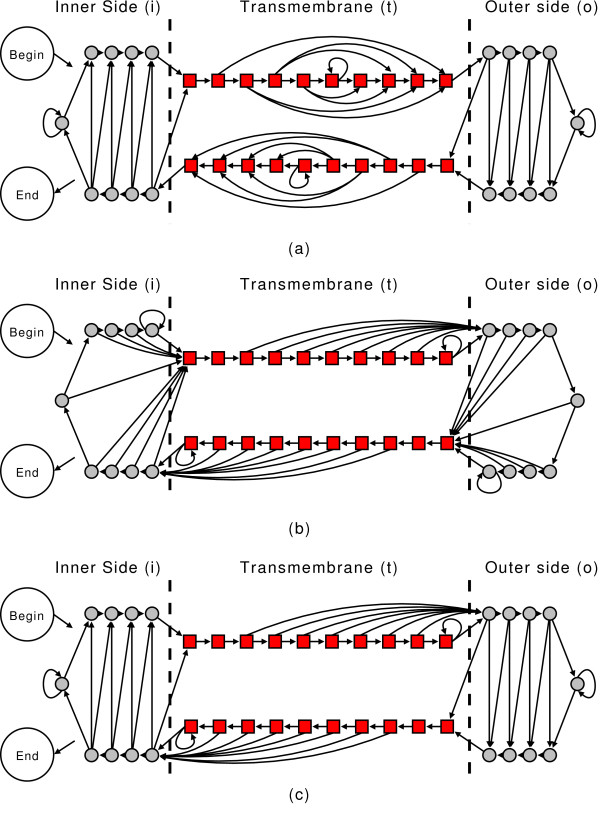
**Three different non-ambigous automata derived from the one depicted in Figure 2**. These automata are designed to have a bijective mapping between the states and the labels (after the corresponding re-labeling of the sequences). In the text they are referred as CRF1 (a), CRF2 (b) and CRF3 (c).

For this reason and for sake of comparison, we designed three other automata (Figure 3a, b and 3c) that have the same number of states but are non-ambiguous in term of state mapping. Then, starting from the experimentally derived labels, three different sets of re-labelled sequences can be derived to train CRFs (here referred as CRF1, CRF2 and CRF3).

All compared methods take as input sequence profile and are bench-marked as shown in Table 1. In the case of non-ambiguous automata of the CRFs, we tested both the Viterbi and posterior-Viterbi algorithms since given the *Viterbi-like *learning of the CRFs it is not *a priori *predictable which one of the two decodings performs better on this particular task. From Table 1 it is clear that assigning the labels according to the posterior-Viterbi always leads to better performance than with the Viterbi (see CRF in Table 1). This indicates that also in other tasks where CRFs are applied, the posterior-Viterbi here described can increase the overall decoding accuracy. Furthermore, the fact that both HMM-B2TMR and GRHCRF perform better than the others, implies that in the tasks where the observed labels may hide a more complex structure, as in the case of the prediction of the Prokaryotic outer membrane proteins, it is advantageous exploiting the ambiguity by taking into consideration multiple concurring paths at the same time, both during training and decoding (see Method section). Considering that underlying grammar is the same, the discriminative GRHCRF outperforms the generative model (HMM-B2TMR). This indicates that the GRHCRF can substitute the HMM-based models when the labeling prediction is the major issue. In order to asses the confidence level of our results, we computed pairwise t-tests between the GRHCRF and the other methods. From the t-test results reported in Table 2, it is evident that the measures of the performace shown in Table 1 can be considered significant with a confidence level greater than 80% (see the most relevant index POV).

**Table 2:** Confidence level of the results reported in Table 1.

**Methods**	**POV**	**Q2**	**C(t)**	**Sn(t)**	**Sp(t)**
GRHCRF vs CRF-1	98.0%	99.5%	99.5%	99.8%	99.5%
GRHCRF vs CRF-2	96.0%	99.5%	99.5%	99.5%	99.5%
GRHCRF vs CRF-3	96.0%	99.5%	99.5%	99.5%	99.5%
GRHCRF vs HMM-B2TMR	80.0%	96.0%	99.0%	98.0%	99.5%

The confidence level on the significance of the differences was computed with a t-test.

For all methods we consider the best results of Table 1.

## Conclusion

In this paper we presented a new class of conditional random fields that assigns labels in agreement with production rules of a defined regular grammar. The main novelty of GRHCRF is then the introduction of an explicit regular grammar that defines the prior knowledge of the problem at hand, eliminating the need of relabelling the observed sequences. The GRHCRF predictions satisfy the grammar production rules by construction, so that only meaningful solutions are provided. In [13], an automaton was included to restrain the solution of a HCRFs. However in that case, it was hard-coded in the model in order to train finite-state string edit distance. On the contrary, GRHCRFs are designed to provide solutions in agreement with defined regular grammars that are provided as further input to the model. To the best of our knowledge, this is the first time that this is described. In principle, the grammar may be very complex, however, to maintain the tractability of the inference algorithm, we restrict our implementation to regular grammars. Extensions to context-free grammars can be designed by modifying the inference algorithms at the expense of the computational complexity of the final models. Since the Grammatical-Restrained HCRF can be seen as an extension of linear HCRF [13,14], the GRHCRF is also related to the models that deal with latent variables such as Dynamic CRFs [22].

In this paper we also test the GRHCRFs on a real biological problem that require grammatical constraints: the prediction of the topology of Prokaryotic outer-membrane proteins. When applied to this biosequence analysis problem we show that GRHCRFs perform similarly or better than the corresponding CRFs and HMMs indicating that GRHCRFs can be profitably applied when a discriminative problem requires grammatical constraints.

Finally we also present the posterior-Viterbi decoding algorithm for CRFs that was previously designed for HMMs and that can be of general interest and application, since in many cases posterior-Viterbi can perform significantly better than the classical Viterbi algorithm.

## Competing interests

The authors declare that they have no competing interests.

## Authors' contributions

PF and CS formalized the GRHCRF model. CS wrote the GRHCRF code. CS and PF performed the experiments. PF, PLM and RC defined the problem and provided the data. CS, PF, PLM and RC authored the manuscript.
